# Altered Ecology of the Respiratory Tract Microbiome and Nosocomial Pneumonia

**DOI:** 10.3389/fmicb.2021.709421

**Published:** 2022-02-10

**Authors:** Ana Elena Pérez-Cobas, Fernando Baquero, Raúl de Pablo, María Cruz Soriano, Teresa M. Coque

**Affiliations:** ^1^Department of Microbiology, Ramón y Cajal Institute for Health Research (IRYCIS), Ramón y Cajal University Hospital, Madrid, Spain; ^2^CIBER in Epidemiology and Public Health (CIBERESP), Madrid, Spain; ^3^Intensive Care Department, Ramón y Cajal University Hospital, Madrid, Spain; ^4^CIBER in Infectious Diseases (CIBERINFEC), Madrid, Spain

**Keywords:** respiratory tract microbiome (RTM), ecology, nosocomial pneumonia, intensive care unit (ICU), dysbiosis, antibiotic resistance genes (ARGs)

## Abstract

Nosocomial pneumonia is one of the most frequent infections in critical patients. It is primarily associated with mechanical ventilation leading to severe illness, high mortality, and prolonged hospitalization. The risk of mortality has increased over time due to the rise in multidrug-resistant (MDR) bacterial infections, which represent a global public health threat. Respiratory tract microbiome (RTM) research is growing, and recent studies suggest that a healthy RTM positively stimulates the immune system and, like the gut microbiome, can protect against pathogen infection through colonization resistance (CR). Physiological conditions of critical patients and interventions as antibiotics administration and mechanical ventilation dramatically alter the RTM, leading to dysbiosis. The dysbiosis of the RTM of ICU patients favors the colonization by opportunistic and resistant pathogens that can be part of the microbiota or acquired from the hospital environments (biotic or built ones). Despite recent evidence demonstrating the significance of RTM in nosocomial infections, most of the host-RTM interactions remain unknown. In this context, we present our perspective regarding research in RTM altered ecology in the clinical environment, particularly as a risk for acquisition of nosocomial pneumonia. We also reflect on the gaps in the field and suggest future research directions. Moreover, expected microbiome-based interventions together with the tools to study the RTM highlighting the “omics” approaches are discussed.

## Introduction

The respiratory tract is an organ system that goes from the nostrils to the lung alveoli. It is divided into the suprathoracic (upper) respiratory tract (URT) and the intrathoracic (lower) respiratory tract (LRT) with a surface of roughly 100 square meters (Ross and Pawlina, 2016). Through the years, several studies have revealed the existence of a resident microbial ecosystem inhabiting airway surfaces: the respiratory tract microbiome (RTM) (Man et al., 2017), is particularly dense in the URT. As the gastrointestinal microbiome (GIM), the RTM constitutes a continuous ecosystem with a longitudinal and transversal gradient of microbial diversity from the nasal and oral cavities to the alveoli (Bassis et al., 2015). However, RTM and GIM differ in biomass, diversity and taxonomic composition. In healthy individuals, the complexity and biomass of the GIM increase from the stomach to the colon, reaching up to 10^10^-10^12^ CFUs/mL (Martinez-Guryn et al., 2019). In the RTM, the oropharynx has a high bacterial density (10^7^-10^8^ CFU/ml), but when moving from the mouth to the lungs, there is a progressive decrease, reaching 10^4^-10^5^ bacterial cells per mL of alveolar intraluminal fluid (Schneeberger et al., 2019). The RTM composition is influenced by the local microbiological growth conditions determined by the nutrient availability, the micro-geography, and the physicochemical conditions such as the mucociliary escalator, oxygen tension, blood flow, pH, temperature, human immune system interactions, and environmental factors (Dickson et al., 2014). The primary source of microorganisms of the RTM is the oropharynx, eventually migrating to the LRT by microaspiration and mucosal dispersion (Bassis et al., 2015; Venkataraman et al., 2015; Dickson et al., 2017). A current model of the ecology of the RTM is known as the “adapted island model of the lung” (Dickson et al., 2014), and it resembles a biogeographical process, which is derived from the equilibrium model proposed by MacArthur and Wilson (1963) of island biogeography (MacArthur and Wilson, 1963). The “adapted model” indicates that the microbial richness in the RTM is based on the balance of immigration (mucosal extension, micro-aspiration) and elimination (ciliary clearance, cough, antimicrobial immunity mechanisms) of species to the lung from the URT. Basically, the RTM structure depends on the rates of stochastic migration, growth, extinction, and epidemic propagation of members of the microbial community, processes that are influenced by anatomical, physiological and clinical factors (Dickson et al., 2014, 2015). This model would help to explain the gradient of microbial diversity, biomass, and complexity decreasing from the oral cavity to the lung. Dickson and colleagues stated that, in health, the lung microbiome is more influenced by microbial immigration and elimination than by local growing conditions. However, local growth conditions are more critical during advanced clinical diseases. This microbiome-based ecological model provides a theoretical framework for pathogen infection, more accurate than the traditional view of pneumonia based on the rapid growth of an invader to a sterile lung.

The bacterial composition of the healthy RTM mainly comprises Firmicutes, Bacteroidetes, and Proteobacteria, which are three of the four predominant phyla in all human site-specific microbiomes. The most abundant genera are *Streptococcus, Prevotella*, or *Veillonella*, which also predominate in the oral microbiome (Araghi, 2020). Only a few studies have characterized other RTM members as fungi, archaea, or viruses (Marsland and Gollwitzer, 2014; Man et al., 2017). With the possible exception of Saccharomycetes (as the genus *Candida*) in the oropharynx, it is difficult to ascertain if at lower (sub-tracheal) levels there is a normal established “respiratory mycobiome,” if the fungi found in the RTM correspond to inhaled environmental fungi, or if a subset of them is less prone to physiological clearance. The healthy respiratory mycobiome is enriched in environmental fungi from the phyla Ascomycota and Basidiomycota, the most common also in the GIT (Tipton et al., 2017). Many fungi correspond to Eremothecium, Systenostrema, Cladosporium genera, and Davidiellaceae family that, together with Saccharomycetes, are also the most common taxa in the GIT. The LRT's virome remains largely unexplored, especially in healthy people. Nonetheless, similar to the gut virome, eukaryotic viruses and many bacteriophages have been described in different studies (Marsland and Gollwitzer, 2014; Mitchell et al., 2016). Some studies point to a function of the respiratory virome in priming and modulating host immune response, as well as the control of other microbial species within the lungs (Mitchell et al., 2016). The factors shaping the virome diversity remain unknown, although the its composition in the respiratory tract appears to be determined by the host health and the presence of specific resident bacterial populations in the bronchi, as it has been suggested in cystic fibrosis (Willner et al., 2009, 2012). A metagenomic study of the virome after lung transplantation based on allograft bronchoalveolar lavage (BAL) samples showed that the respiratory tracts of lung transplant recipients were enriched in complex populations of anelloviruses (Young et al., 2015). Viral loads have also been correlated with bacterial dysbiosis which influences transplant outcomes and suggests that viral-bacterial interactions are critical for microbiome-immune system equilibrium and, thus, to its physiology. Moreover, the high abundance of bacteriophages in the RTM deserves in-depth research to determine if they play similar roles as those observed in the GIM, namely, controlling microbial homeostasis (blooms, microbial composition, diversity, metabolism, and facilitating horizontal gene transfer (HGT) (Maurice, 2019; Sutton and Hill, 2019). Methanogenic archaea have been detected in the nasal, GIT, skin, and lung microbiomes, and the phylum Woesearchaeota is apparently associated with the lungs (Koskinen et al., 2017). However, besides a few descriptive studies, the function of the non-bacterial fraction of the RTM deserves to be further analyzed.

The RTM ecology is an emerging research area gaining attention since several studies showed the beneficial role of microbial inhabitants in the stimulation of the immune system and the protection against pathogen (colonization resistance, CR), as it has been extensively documented for the intestinal microbiome (examples reported by Thibeault et al., 2021). The microbiome homeostasis can be transiently disturbed, for instance, by antibiotic usage, application of medical equipment (intubation, ventilators), transient abnormal events (aspiration), or particular diseases (viral diseases might reduce the effectiveness of the bronchial clearance escalator) allowing some pathogens to overgrow and boosting the colonization and, ultimately to cause infection at LRT. Most of the studies related to the RTM have been focused on particular chronic respiratory diseases, such as microbial populations colonizing the lung in cystic fibrosis (reviewed in O'Toole, 2018; Françoise and Héry-Arnaud, 2020). In this disease, the respiratory tract hyper viscosity promotes polymicrobial proliferation and dysbiosis along the respiratory tract (Françoise and Héry-Arnaud, 2020). The most frequent bacteria associated with cystic fibrosis chronic colonization are *Pseudomonas aeruginosa, Staphylococcus aureus, Stenothrophomonas maltophilia, Burkholderia*, and *Pandoraea*, which frequent co-colonize. However, oral-cavity-related microorganisms are also present as *Actinomyces, Fusobacterium, Gemella, Granulicatella, Neisseria, Porphyromonas, Prevotella, Rothia, Streptococcus, Haemophilus*, or *Veillonella*. Interestingly, predator bacteria in the RTM-LRT as *Bdellovibrio* and *Vampirovibrio*, and bacterial parasites of the phylum Parcubacteria can be found in cystic fibrosis patients (Caballero et al., 2017), This last work suggests a possible role of predator bacteria from the RTM in controlling chronic colonization by pathogens in early colonization stages of cystic fibrosis, highlighting the involvement of the RTM bacterial fraction in fighting pathogenic bacterial populations.

Here, we present our perspective regarding research in altered RTM ecology in the clinical environment, increasing the probability for acquiring nosocomial pneumonia in high-risk hospital areas as ICUs, and discuss recent advances on the topic. We also discuss the gaps in the field, the future of microbiome-based interventions to prevent and treat nosocomial pneumonia, and the contribution of “omics” approaches to understanding the role of the RTM dynamics in the onset of respiratory infections.

## The Respiratory Microbiome Ecology in Nosocomial Pneumonia

Acute LRT infections, pneumonia or exacerbations of chronic bronchitis, are the leading cause of mortality and morbidity worldwide (Troeger et al., 2018). In the elderly, the progression of pneumonia happens fast, with poor prognosis, primarily associated with hospitalization, and high mortality rate frequently in ICUs (Henig and Kaye, 2017). Nosocomial pneumonia, a lung alveoli infection that mostly bacteria, but also viruses, or fungi can cause comprises hospital-acquired and ventilator-associated pneumonia and is of significant public health concern since it is frequently caused by multidrug-resistant (MDR) pathogens acquired within the hospital environment (Denys and Relich, 2014). Ventilator-associated pneumonia is the first cause of nosocomial infection in mechanically ventilated patients and the second most common one at intensive care units (ICU), with long-term infections often caused by MDR bacteria (Kalanuria et al., 2014). This scenario has worsened during the COVID-19 pandemic due to the dramatic increase in SARS-CoV-2 secondary infections and the over-use of antibiotics, leading to a remarkable increase of MDR infections and antibiotic resistance gene (ARGs) transmission (Feldman and Anderson, 2021; Langford et al., 2021; Soriano et al., 2021). The microbiome's contribution to innate and adaptive immune responses suggests that a healthy microbiome may be a factor contributing to a lower case fatality ratio from COVID-19 (Khatiwada and Subedi, 2020; Janda et al., 2021). On the other hand, microbiome dysbiosis could be associated with poor immune response and worse disease outcomes (Stavropoulou et al., 2021). The field is new and available studies regarding the COVID-19 microbiome have reported contradictory results (Yamamoto et al., 2021). One of the factors leading to contradictions seems to be confounders such as mechanical ventilation in ICU patients, altering the RTM community structure, including the abundance of oral taxa previously associated with COVID-19 (Lloréns-Rico et al., 2021). Studies sampling the infected lungs directly through bronchoscopy have proven to be the most accurate so far for identifying microbiological and immunologic alveolar signatures associated with SARS-CoV-2 infection (Dickson, 2021). A work based on 142 patients who underwent bronchoscopy showed that clinical outcomes could be partly explained by alveolar viral abundance, and colonization by URT microbiome-derived bacteria (*Mycoplasma salivarium*), and poor adaptive immune response (Sulaiman et al., 2021).

An ecological model of pneumonia proposes that the RTM equilibrium is displaced toward a state of dysbiosis characterized by low microbial diversity, high microbial burden, and host inflammatory response (Dickson et al., 2014). Whether dysbiosis is the cause or the effect of the pneumonic disease remains obscure. In fact, the role and dynamics of the RTM microbiome in critical patients have been scarcely investigated, and therefore microbiome-directed interventions have not been included in clinical guidelines. Certainly, RTM disruption can be influenced by physiological circumstances altering the local ecology (Dickson et al., 2016), including: (1) inflammation, alteration of metabolites profile, and weakened local defense mechanisms by infections (i.e., virus), favoring bacterial stochastic migration and growth (Madhi and Klugman, 2004; Gu et al., 2019); (2) abnormal chemical composition of the bronchi, as in cystic fibrosis (Goldman et al., 1997); (3) anatomical abnormalities and other conditions leading to obstruction, as in bronchiectasis, cystic fibrosis, chronic obstructive lung disease, pulmonary edema (which can be secondary to sepsis), or lung cancer (Hogg et al., 1970; Peteranderl et al., 2017; Vengoechea et al., 2019); (4) external harmful conditions, as long-term exposure to cold, leading to mucosal vasoconstriction in the respiratory tract mucosa and suppression of immune responses, or allergenic drivers for asthma (Depner et al., 2017); (5) increased bacterial migration (i.e., thorough aspiration of gastric fluids, altered oral microbiome, intubation, unconsciousness, supine position), and decreased elimination process (unconsciousness, intubation, sedatives, head raised, impaired mucociliary clearance) (Dickson, 2016); (6) underlying diseases and medical interventions (i.e., therapeutical immunosuppression, intubation, mechanical ventilation, or long-term antibiotic therapy) favoring colonization of opportunistic pathogens.

**Figure 1:**
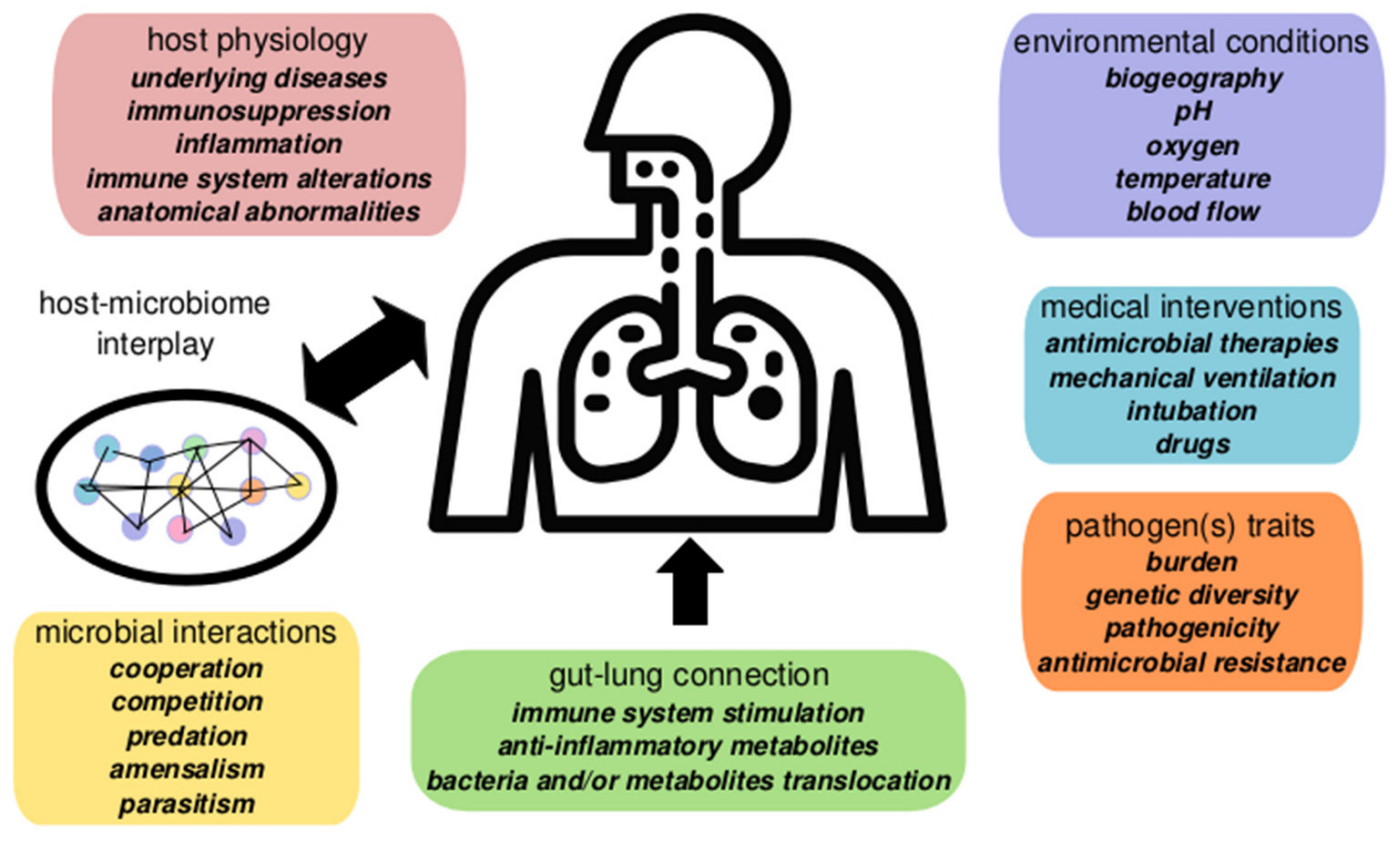
Summary of factors involved in nosocomial pneumonia dynamics in critical patients. Pneumonia evolution and outcome are influenced by host interplay with the respiratory microbiome determined by the microenvironment, microbial interactions, and host physiology. The gut microbiome contributes to pneumonia responses via the gut-lung axis (gut microbiome changes alter systemic and lung physiology). Environmental conditions and clinical factors, as mechanical ventilation, or antibiotics, also play a significant role in disease, altering the microbiome ecology and the pathogen(s) population dynamics.

Nosocomial pneumonia can be endogenous, caused by opportunistic pathogens as *Streptococcus pneumoniae* or *Haemophilus influenzae*, which are part of the healthy human RTM. This pneumonia generally appears soon after admission. However, late-onsent cases of pneumonia development frequently involve hospital-acquired MDR pathogens, including *P. aeruginosa*, Enterobacteriaceae producing extended-spectrum-beta-lactamases (ESBL), and/or carbapenemases, and methicillin-resistant *Staphylococcus aureus* (MRSA), which are often selected and spread in the ICU environment (Denys and Relich, 2014). The pathogen-centric view of infectious diseases has recently moved toward an ecological perspective based on host-pathogen-microbiome-environment interactions (Wu and Segal, 2018). In this context, nosocomial pneumonia represents a complex scenario where several factors may play a role in the onset and the outcome of the infection (Figure 1).

## Effect of Antibiotics and Other Medical Interventions in the RTM of Critical Patients

The intense use of broad-spectrum antibiotics in the ICU favors the selection of resistant bacteria (i.e., *P. aeruginosa*, MRSA, multi-resistant Enterobacterales) or paranasal viruses established in the oral-nasopharyngeal cavity, which can colonize and eventually cause respiratory infections (Bert and Lambert-Zechovsky, 1996; Volakli et al., 2010). In this respect, RTM contribution to pneumonia development and severity, or the eventual implication in the transmission of antimicrobial resistance (AMR) remains poorly analyzed. As well as systemic alteration through depletion of the GIT microbiome, antibiotic usage locally affects the RTM during respiratory infections, especially in ventilated-associated pneumonia (Fernández-Barat et al., 2020). The administration of antimicrobial drugs transiently disrupts the RTM homeostasis and thus, impair CR capacity (Fernández-Barat et al., 2020; Pettigrew et al., 2020). It results in local selection and overgrowth of resistant pathogenic and opportunistic species, increasing the chances of co-infections. However, the effects of doses, the length of therapy, the route of administration, and the spectrum of the antibiotics in the ecology of RTM have not been fully characterized, especially considering that classic antibiotic therapy of respiratory tract infections is directed to pathogen eradication (Dagan et al., 2001; Ball et al., 2002). Further research on the influence of common antibiotics used in ICU medicine on RTM composition and resistome is necessary to implement more efficient interventions.

Moreover, the ARG reservoirs and transmission mechanisms in the human respiratory microbiome remain poorly characterized, particularly in ICU patients (Kim and Cha, 2021). An interesting study based on metatranscriptomics and 16S rRNA-gene sequencing revealed that response to influenza infection could indirectly affect the expression of ARGs in the respiratory tract by impacting the microbiome diversity and overall the microbial gene transcription (Zhang et al., 2020). In this connection, *S. pneumoniae*, a common respiratory pathogen, can kill closely related bacteria (a phenomenon known as fratricide) by competence-activated killing factors (bacteriocins). The killing leads to HGT by acquiring exogenous DNA from the dead cells, including ARGs (Veening and Blokesch, 2017). Another exciting example related to ARGs acquisition is the case of *Acinetobacter* species which are MDR pathogens causing respiratory infections. Cooper and colleagues showed a predator-prey dynamics where *Acinetobacter baylyi* using its type VI Secretion System (T6SS), lyses and acquires genes through HGT from neighboring *Escherichia coli* cells, including ARGs (Cooper et al., 2017). Similar to species as *A. baylyi* or *Vibrio cholerae* a T6SS-dependent killing of neighboring bacterial cells promoting the uptake of foreign DNA could lead to antibiotic resistance or virulence in other bacteria associated with respiratory infections as *Pseudomonas* or *Ralstonia* (Ringel et al., 2017). These strategies contribute to the fitness of the stronger competitor or the predator bacteria by acquiring beneficial adaptive traits, including the uptake of plasmids and ARGs.

Among medical ICU interventions, ventilation is one of the main factors shaping the composition of the respiratory microbiome. The significant variability between the few available mechanically-ventilated patient-based studies is a challenge for establishing conclusions (Fromentin et al., 2021). As expected, the microbiome in ICU ventilated patients is more disturbed than in non-ventilated individuals as it reflects the decrease of the alpha-diversity with the duration of mechanic ventilation and the frequent overgrowth of one or a few species (Kelly et al., 2016; Zakharkina et al., 2017). Besides differences in the alpha diversity, the RTM in mechanically ventilated patients shows differences in the composition and the abundance of microbial entities when compared to controls (Emonet et al., 2019). Emonet and colleagues identified early markers associated with the development of ventilator-associated pneumonia (Emonet et al., 2019). Dickson et al. performed a prospective, observational cohort study in critically ill patients with mechanical ventilation. The authors found that bacterial burden and composition of the RTM predict the ICU-patient outcome (Dickson et al., 2020). More studies of this nature would significantly improve the prevention and diagnosis of ventilator-associated pneumonia by identifying the risk, severity, and evolution of RTM markers. The diagnosis, prognosis, and therapies should consider all these factors to adopt a precise medical approach to manage respiratory infections.

## Gut-Lung Axis in Respiratory Infections

The microbial gut-lung axis (cross-talk between the intestinal and the respiratory microbiotas) seems to work through systemic dissemination of beneficial anti-inflammatory and antimicrobial metabolites, such as short-chain fatty acids **(**SCFAs) or by the stochastic propagation of bacteria (Dumas et al., 2018). Mice studies have proved that the GIM is protective against respiratory infection through pulmonary immune system stimulation (Budden et al., 2017). Gut microbiome perturbations are linked with altered immune responses and homeostasis in the airways (Dang and Marsland, 2019). Note that gut microbiota dysbiosis generally results in the overgrowth of subpopulations of Proteobacteria (e.g., some genus/families of Gammaproteobacteria) present as minority populations in the gut, which frequently colonize the upper intestine, thus increasing the chances of reaching the respiratory tract. This often occurs under antibiotic therapy and underlying patient conditions such as obesity and under-nutrition. In cystic fibrosis, changes in the dominance of certain species in the GIM are followed by an increase of the same species in the respiratory tract (Madan et al., 2012).

The gut-lung axis might become harmful in circumstances of famine and during critical illness. In the ICU, the customary use of anti-acid therapy (facilitating stomach-esophageal-mouth-tracheal colonization) might contribute to gut-respiratory transmission. The abdominal cavity (peritoneum) and the thoracic cavity (pleura) are neighbor spaces separated by a dynamic and not totally hermetic membrane (diaphragm), and then pneumonia is frequently associated with peritonitis (Muniz et al., 2015). Recently, patients with COVID-19, besides the evident pulmonary infection, have been reported to present gastrointestinal symptoms, pointing to the gut-lung axis as a strategy to prevent and treat the disease (He et al., 2020). The basis and mechanisms of the gut-lung axis in respiratory infections needs to be addressed by combining animal model and clinical analyses. Longitudinal sampling of the gut and the RTM from ICU patients would provide much knowledge on directionality and connection among both systems. Also, whole-genome comparisons of species present in the GIM and RTM could cast some light on connectivity of both ecological spaces.

## “The Black Hole” of the RTM in Health and Disease

The RTM field is in its infancy, and the role of the RTM for host physiology in health and disease is far from being understood. More than knowledge gaps or pieces of information, there is a “black hole” regarding the ecology of the RTM and the consequences derived from its disturbances. One of the main gaps in the field is the poor knowledge of the healthy RTM baseline data, especially regarding the LRT. The area involved in the nutrients acquisition, which is the primary function of the gastrointestinal tract, has evolved (acidity, bile, peristalsis) to contain a minimum microbial load. Similarly, the area involved in oxygen acquisition by the respiratory tract has evolved to be very poorly colonized (mucociliary escalator, cough reflex). Whether a normal indigenous RTM in the LRT persists over time or is constant community immigration from the URT is a matter of debate and challenge. Until the development of high-throughput sequencing approaches, the LRT colonizers in the absence of infection have been disregarded. This fact was primarily due to the failure to grow lung microbes in routine microbiological cultures (Bos and Kalil, 2019). Moreover, collecting clinical samples suitable for describing the LRT microbiome, such as tracheal aspirates or BALs, is an invasive procedure that makes it quite challenging for performance on non-hospitalized healthy individuals and does not prevent oropharyngeal contamination.

Like any microbial ecosystem, the RTM functions through microbial interactions taking place in the tract niches with a strong influence on the host through the immune system, and the environment, especially in a body part in constant air exchange. Most of the ecological processes that may occur in this microbiome, including diversity dynamics, stability, resistance, resilience, or microbial interactions (i.e., cooperation, competition, amensalism) are poorly understood. Furthermore, the identification of “keystone” microorganisms (those with a substantial impact on the performance of ecosystems that, when affected, have a significant effect on the whole arrangement as key-nodes in a network) and their microbial interactions (network's edges), generally involving mechanisms such as metabolite exchange, metabolite conversion, signaling, or genetic exchange, remain largely unknown. Identifying the RTM composition, the microbial interactions, and the mechanisms involved in CR are becoming relevant to define a “healthy microbiome” along the respiratory tract and to understand the impact of clinical and environmental factors that lead to dysbiosis. The development of accurate, personalized microbiome-based treatments would significantly remodel the management of patients with respiratory diseases.

The detection of microbial eukaryotes (fungi and protozoa), archaea, and viruses have been neglected in RTM research. However, through the application of “omics,” the RTM composition of non-bacterial microorganisms is starting to be described (Koskinen et al., 2017; Xu et al., 2017; Pérez-Cobas et al., 2020a). The mycobiome and virome should be considered a high priority due to the contribution of viruses and fungi in the development of respiratory infections (Wu and Segal, 2018). In pneumonia and other infectious diseases, intra- (bacteria-bacteria, fungi-fungi) and inter-kingdom interactions (bacteria-fungi, bacteria-viruses) seem to influence the severity of the infections and might have a role in host-microbiome equilibrium restoration after disease remission (Molyneaux et al., 2013; Pérez-Cobas et al., 2020a). Since the microbial ecosystem works as a network of connections, it is wise to consider bacteria and their interactions with other kingdoms to treat infections. On the one hand, that would indirectly allow targeting infections through their co-dependent partners disrupting the whole dysbiotic consortium. On the other hand, knowing the network of beneficial partners would also make it possible to stimulate a set of microorganisms that might contribute to accelerating health recovery through microbiome restoration.

## Microbiome-Based Interventions to Treat Nosocomial Respiratory Infections

As previously stated, hospitalized patients are generally under antibiotic therapy, especially critical ones in the ICUs with mechanical ventilation, long-term stays, and immunosuppression. Thibeault et al. recommend a personalized and rational use of antibiotics, the monitorization of the treatment, and frequent and continuous reevaluation of the therapeutic regimen as a strategy to overcome the effect of antimicrobials on the microbiota (Thibeault et al., 2021). A good choice of pathogen-focused antibiotics should effectively kill the pathogen while causing minimized damage to the microbiota. Research on the effect of the clinical most used antibiotic combinations in the RTM would improve the antibiotics therapy choices in ICU patients.

Probiotics (administered living bacteria beneficial for health) have been applied to restore the gut microbiome in several conditions associated with antibiotics treatments, although studies of its efficacy have yielded heterogeneous but moderately positive effects (Hempel et al., 2012; Goldenberg et al., 2018). They can be administered combined with prebiotics (diet components that positively stimulate the microbiome leading to benefit for host physiology), the combination being called synbiotics. Preliminary studies suggest a positive effect of probiotics and synbiotics and high safety in nosocomial and ventilator-associated pneumonia patients. These studies correspond to different and heterogeneous clinical trials and, thus, are not conclusive (Liu et al., 2012; van Ruissen et al., 2019; Batra et al., 2020) which makes it necessary to increase the efforts in this area. Targeting the respiratory microbiome directly through the airways (i.e., inhalation) would be helpful to progress in the prevention and therapy of nosocomial pneumonia.

Fecal microbiota transplantation (FMT) is a promising approach that has been successfully applied to restore the microbiome of patients under recurrent *Clostridium difficile* (Juul et al., 2018). Some clinical studies on *C. difficile* showed that FMT, along side the microbiome equilibrium recovery, reduces intestinal colonization by MDR bacteria (Millan et al., 2016; Saha et al., 2019; Ghani et al., 2020). Also, mouse model studies showed that intestinal and URT microbiome restoration could re-establish the pulmonary immune defenses against bacterial and viral infections through mechanisms as enhancing primary alveolar macrophage function (Schuijt et al., 2016; Brown et al., 2017; Sencio et al., 2021). The set-up and application of this methodology to target the respiratory microbiome could significantly improve patient response to treatments and impede AMR transmission. FMT could restore the protection capacity against nosocomial infections by MDR bacteria, a current major problem in the ICU environment. Besides probiotics and FMT the possibility of a cocktail of known species involved in CR against pathogens, immune system homeostasis, and respiratory microbiome ecology restoration (keystone species) would represent a milestone in targeted microbiome interventions.

As the FMT stimulation of the intestine activates the immune lung responses, another way to cure respiratory infections via the gut-lung axis is by stimulation of the production of gut microbe-derived metabolites, such as the SCFAs or polyamines that have positive systemic effects (Rooks and Garrett, 2016). Various studies have shown that SCFAs are known to stimulate the immune system protecting against viral and secondary bacterial respiratory infections (reviewed in Sencio et al., 2021). Animal and clinical studies using supplementation with these metabolites or stimulation of the growth of producers could contribute to preventing and better fighting against respiratory infections.

Although still in a very early stage, the bacteriophage-based therapies seem very promising to fight pneumonia caused by MDR bacteria (Wunderink, 2019). A recent report showed high levels of bacterial clearance after the administration of an intravenous and aerosol four-phage cocktail to a patient with in a multilobe cavitary resistant *Pseudomonas* infection (Maddocks et al., 2019). Another study shows how an intravenous cocktail of four bacteriophages was as effective as teicoplanin in improving survival and decreasing bacterial load in a mouse model of pneumonia by MRSA (Prazak et al., 2019). Another approach is a vaccination to reduce the antimicrobial resistance burden by targeting bacterial pathogens that can be resistant. When focused on lung pathogens, vaccination against S. pneumoniae and H. influenzae to reduce high mortality bacterial complications like pneumonia or sepsis lead to a reduction in antibiotic-resistant lineages (Ginsburg and Klugman, 2017). Bacteriophage-based pathogen elimination and vaccination act against specific agents and are partially effective when co-infections are in a dysbiotic microbiome environment. A disturbed microbiome is ineffective in stimulating specific immune system pathways involved in protections against infections.

The restoration of the immune system capacity through local stimuli is a novel prophylactic strategy that has been successfully tested in mice. Specifically, direct applications of IgA, IL-22, or antibodies that block IL-10 help protect microbiota-depleted mice from bacterial pneumonia (reviewed in Thibeault et al., 2021). Robak and colleagues showed that depletion of microbiota by antibiotics inhibited TLR-dependent production of a proliferation-inducing ligand (APRIL), leading to a deficiency in a secondary IgA in mice and human ICU patient's lungs (Robak et al., 2018). Furthermore, since the IgA contributes to defense against *P. aeruginosa*, the authors also demonstrated that after transnasal direct application in antibiotic-treated mice, *P. aeruginosa*-binding IgA provided resistance to infection. Further research combining targeted approaches as bacteriophage therapy or vaccination with restore-based ones as probiotics or prebiotics is promising.

## Future Directions: Next Steps in the RTM Research

The “omics” have transformed research in clinical microbiology and infectious diseases, contributing to understand microbiome ecology in health and disease (Relman, 2011). The RTM is generally approached from samples such as sputum, tracheal aspirates, or BALs that generally present low biomass, high host contamination, frequent manipulation-derived contaminations, making marker genes sequencing a suitable option (Perez-Cobas and Buchrieser, 2019). So far, the most applied culture-independent methodology to analyze microbial communities' diversity and composition is marker gene sequencing such as 16S rRNA for archaea and bacteria and internal transcribed spacer (ITS) for fungi (Fromentin et al., 2021). Marker genes derived data provided a vision on the relative abundance of the taxa; however, the absolute abundance values are often not estimated. Therefore, the marker genes approach combined with quantification methods of the selected fractions such as qPCR, spikes, or droplet-digital PCR will be relevant to have a more accurate picture of the microbiome evolution during disease, considering not only the relative abundance but the biomass dynamics (Dickson et al., 2020; Pérez-Cobas et al., 2020a).

On the other hand, the omics field is evolving toward the shotgun metagenomic sequencing, which allows characterizing whole metagenomes, reaching a strain level of resolution, and exploring the diversity of non-bacterial diversity such as eukaryotes and viruses (Pérez-Cobas et al., 2020b). However, the low microbial biomass and the enrichment in human cells of respiratory samples complicate the application of metagenomics approaches. Recent-developed protocols deplete human and extracellular DNA from low-biomass abundance samples, yield optimized microbiome profiles, and allow quantifying live microbial load (Marotz et al., 2018, 2021; Nelson et al., 2019). Respiratory clinical samples enriched in microbial cells will enable the application of other omics more focused on understanding the RTM's metabolic, enzymatic, and functional capacities, including meta-metabolomics, metaproteomics, or metatranscriptomics. As previously stated, AMR is a major problem to treat critical patients with pneumonia, and the contribution of the RTM to the acquisition, development, or maintenance of AMR is practically unknown. Besides shotgun metagenomics, future studies should include specific characterization of the resistome and mobilome based on novel targeted-metagenomics (Lanza et al., 2018). More investigation focusing on bacteriophages that are frequent carriers of resistances is needed to fully understand ARGs dynamics and transmission. For instance, a method set-up for skin and wound swabs allows several hundred-fold enrichment of viral DNA, improving viromics performance (Verbanic et al., 2019).

Culture-independent approaches will contribute to obtain the RTM whole picture of diversity, composition, metabolism, expression, ARGs, shedding light on its ecology and physiological role. Together with clinical and environmental variables, this biological information would allow identifying biomarkers of illness prognosis and severity, leading to significant improvement of diagnostic tools, and personalized therapeutic regimens. To complement and test omic-derived data, culturomics, *in vitro* studies, animal models, and longitudinal human clinical studies are necessary to understand the lung microbiome's ecology in infectious diseases and further develop microbiome-based therapies.

## Data Availability Statement

The original contributions presented in the study are included in the article/supplementary material, further inquiries can be directed to the corresponding authors.

## Author Contributions

The manuscript was written by AEP-C, FB, TC, RP, and MCS. All authors listed have made a substantial, direct, and intellectual contribution to the work and approved it for publication.

## Funding

Lab research in this topic was supported by grants funded by Fundación Ramón Areces (BIOMETASEP), and the Instituto de Salud Carlos III (PI18/1942) co-funded by the European Regional Development Fund (ERDF, A way to achieve Europe). Also supported by InGEMICS-CM (B2017/BMD-3691), funded by Comunidad de Madrid (Spain) and CIBER (CIBER in Infectious Diseases, CIBERINFEC CB21/13/00084) and (CIBER in Epidemiology and Public Health, CIBERESP; CB06/02/0053), integrated in the Spanish 2013-2016 I+D+i State Plan and funded by Instituto de Salud Carlos III. AEP-C is recipient of a Grant for the Attraction of Talent within the Comunidad de Madrid (Grant No. 2019-T2/BMD-12874).

## Conflict of Interest

The authors declare that the research was conducted in the absence of any commercial or financial relationships that could be construed as a potential conflict of interest.

## Publisher's Note

All claims expressed in this article are solely those of the authors and do not necessarily represent those of their affiliated organizations, or those of the publisher, the editors and the reviewers. Any product that may be evaluated in this article, or claim that may be made by its manufacturer, is not guaranteed or endorsed by the publisher.
